# Bone Health in Children and Adolescents with Type 1 Diabetes: Optimizing Bone Accrual and Preventing Fractures

**DOI:** 10.3390/nu17152400

**Published:** 2025-07-23

**Authors:** Neriya Levran, Einat Shalev-Goldman, Yael Levy-Shraga

**Affiliations:** 1 Pediatric Endocrinology and Diabetes Unit, The Edmond and Lily Safra Children’s Hospital, Sheba Medical Center, Tel Hashomer, Ramat-Gan 5262000, Israel; 2 The Institute of Biochemistry, Food Science and Nutrition, The Faculty of Agriculture, Food and Environment, The Hebrew University of Jerusalem, Rehovot 7610001, Israel; 3 Division of Nutrition Unit, Sheba Medical Center, Tel Hashomer, Ramat-Gan 5262000, Israel; 4 Faculty of Medical and Health Sciences, Tel Aviv University, Tel Aviv 6997801, Israel

**Keywords:** type 1 diabetes, bone health, fractures, nutrition, physical activity

## Abstract

Children and adolescents with type 1 diabetes (T1D) often experience abnormalities in bone health. Studies have consistently demonstrated that youth with T1D have lower bone mineral density (BMD) compared to their healthy peers. Additionally, children with T1D show impaired bone microarchitecture and reduced bone turnover. These factors collectively contribute to an increased risk of fractures across the life span of this population. To optimize bone accrual and reduce fracture risk, several strategies can be employed during childhood and adolescence. First, maintaining good glycemic control is critical, as poor glycemic control has been associated with lower BMD and an increased risk of fractures. Second, specific nutritional recommendations can help improve bone health, including a balanced diet, adequate calcium and vitamin D intake, and careful monitoring of both macronutrient and micronutrient intake. Third, regular physical activity plays a vital role. A systematic review and meta-analysis have shown that youth with T1D are generally less physically active, more sedentary, and have lower cardiorespiratory fitness levels than their non-diabetic peers. This review emphasizes targeted strategies aimed at optimizing skeletal health in the pediatric population with T1D, with a particular focus on the critical roles of glycemic control, nutritional adequacy, and regular physical activity. These modifiable factors may contribute to the reduction of fracture risk across the life span in individuals with T1D.

## 1. Introduction

**Figure 1:**
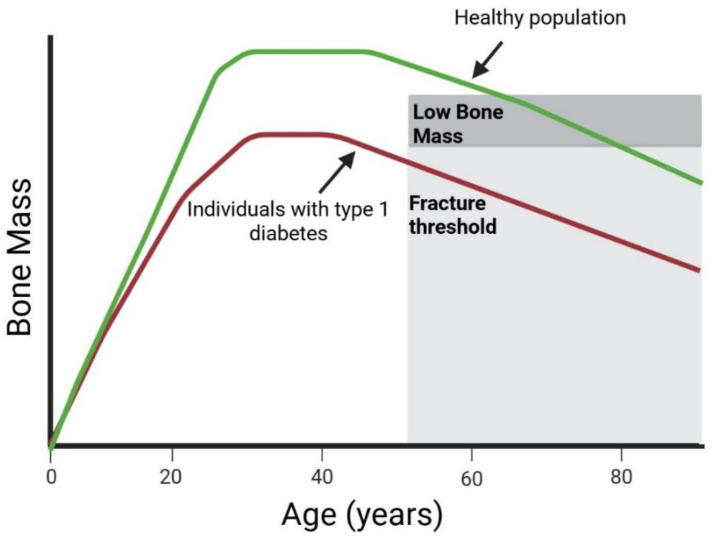
Peak bone mass development and type 1 diabetes. Adapted with permission from Weaver et al. [9], licensed under CC BY-NC 4.0. Some modifications were made to the original figure. http://creativecommons.org/licenses/by-nc/4.0/, accessed on 18 July 2025.

Impaired skeletal health in type 1 diabetes (T1D), once a largely underrecognized complication, has recently received increased attention, as emphasized in the 2022 Clinical Practice Consensus Guidelines from the International Society for Pediatric and Adolescent Diabetes (ISPAD) [1]. Individuals with T1D often exhibit reduced bone mineral density (BMD) [2,3], compromised bone microarchitecture [3] and decreased levels of bone turnover markers [4]. Bone loss begins at disease onset, often during childhood, leading to suboptimal bone accrual and attainment of peak bone mass [5]. This skeletal deficit may predispose individuals with T1D to a persistently elevated risk of fractures, particularly in later adulthood (Figure 1) [6,7,8].

### 1.1. Mechanism of Bone Fragility in T1D

The underlying pathophysiological mechanism through which T1D impacts skeletal integrity remains incompletely elucidated. It is presumed to be multifactorial in nature, involving complex interactions between metabolic, hormonal, and inflammatory pathways [10,11,12,13]. Hyperglycemia exerts direct effects on bone by inhibiting osteoblast maturation and differentiation, as evidenced by an experiment with rat bone marrow stromal cells cultured under osteogenic conditions [14]. It also promotes osteocyte senescence and apoptosis, thereby impairing mechanosensing and stress adaptation, as demonstrated in a diabetic mouse model [15]. Advanced glycation end products (AGEs) are biochemical compounds formed through the interaction of reducing sugars with amino groups, a process that is enhanced under hyperglycemic conditions [16]. AGEs alter collagen cross-linking and negatively affect the differentiation and function of osteoblasts [17].

Moreover, hyperglycemia induces the release of pro-inflammatory cytokines, such as IL-1, IL-6, and TNF-α, which impair osteoblast differentiation and survival while promoting osteoclast activity; thereby, disrupting bone remodeling in favor of resorption [18,19].

Additionally, T1D is associated with dysregulation of the growth hormone (GH)/insulin-like growth factor-1 (IGF-1) axis, characterized by reduced circulating IGF-1 levels and hepatic GH resistance, likely driven by portal insulin deficiency [20]. These endocrine disturbances impair bone mineral accrual and hinder the attainment of peak bone mass. With disease progression, vascular complications and increased oxidative stress increasingly contribute to the development of skeletal fragility [21].

### 1.2. Bone Mineral Density in Pediatric Patients with T1D

Impaired bone development in children with diabetes was first documented in 1927 [22]. Over the past two decades, a growing body of evidence has investigated BMD in children and adolescents with T1D, highlighting a consistent pattern of skeletal deficits in this population.

A comprehensive meta-analysis by Loxton et al. evaluated BMD in pediatric patients (age < 20 years) with T1D using a range of diagnostic modalities, including dual-energy X-ray absorptiometry (DXA), peripheral quantitative computed tomography (pQCT), and quantitative ultrasound (QUS) [2]. The analysis included 46 studies encompassing 6468 participants (2617 with T1D and 3851 age-matched controls), with a mean age of 12.6 ± 2.3 years. The results demonstrated a significantly lower BMD in youth with T1D compared to controls. Specifically, the mean difference in lumbar spine BMD z-score measured by DXA was −0.46 (95% CI: −0.75 to −0.18; *p* = 0.001), and in total body BMD z-score, −0.70 (95% CI: −1.02 to −0.38; *p* < 0.001).

Another large-scale meta-analysis by Zheng et al. examined bone mineral content (BMC) and BMD in children and adolescents with T1D (mean age ≤ 18 years; age range: 1–24 years) across 42 studies, 36 of which were included in the meta-analysis [3]. This analysis incorporated data from 2222 individuals with T1D and 2316 non-diabetic controls. Standardized mean differences (SMDs) with 95% confidence intervals (CIs) were calculated using Hedges’ g, based on groups means or Z-scores, standard deviations (SDs), and sample sizes. Raw means were preferred to ensure consistency across studies. The SMD were −0.30 (95% CI: −0.50 to −0.11) for total body BMD, −0.20 (95% CI: −0.32 to −0.08) for lumbar spine BMD, and −0.30 (95% CI: −0.48 to −0.13) for bone mineral apparent density. Older age and longer disease duration were associated with more pronounced deficits in total body BMD. Notably, the deficits in BMD Z-scores reported in the meta-analysis by Loxton et al. were more pronounced than the SMDs in BMD observed in the analysis by Zheng et al., likely reflecting differences in the quantitative methodologies employed [3]. Nevertheless, both meta-analyses consistently demonstrate reductions in BMC and BMD in pediatric populations with T1D, potentially contributing to an increased lifetime risk of fractures.

### 1.3. Bone Microarchitecture

BMD is a key determinant of bone strength and fracture risk. However, other important factors influence bone strength, including bone mineralization, bone turnover, cortical bone macrogeometry, and bone microarchitecture [23,24]. The latter can be evaluated using pQCT and high-resolution pQCT. A meta-analysis comprising eight pQCT and three HR-pQCT studies reported several statistically significant deficits in individuals with T1D compared with controls quantified by standardized mean differences (SMD) [3]. Among the observed deficits were distal radius trabecular density (SMD −0.38; 95% CI: −0.64 to −0.12), distal radius trabecular bone volume fraction (SMD −0.33; 95% CI: −0.56 to −0.09), distal tibia trabecular density (SMD −0.35; 95% CI: −0.51 to −0.18), distal tibia bone volume fraction (SMD −0.37; 95% CI: −0.60 to −0.14), and distal tibia trabecular thickness—(SMD: −0.41; 95% CI: −0.67 to −0.16). These trabecular deficits are clinically relevant, as compromised trabecular bone contributes to increased fracture risk [25,26]. In contrast, no significant differences were observed in the cortical area, thickness, density, or porosity at the distal radius or tibia between children with T1D and healthy controls. These findings are consistent with those of another meta-analysis, which reported comparable cortical density between the two groups [2].

### 1.4. Bone Turnover Markers

Bone remodeling is a dynamic and lifelong physiological process governed by the balanced activity of osteoblasts and osteoclasts, particularly crucial during the rapid growth of childhood and adolescence [27]. While BMD remains the standard clinical tool for assessing bone mass, it provides limited information on the metabolic activity and quality of bone, especially in pediatric populations and in the context of chronic metabolic conditions [27,28]. Bone turnover markers (BTMs)—biochemical byproducts of bone formation and resorption—offer valuable real-time insight into skeletal metabolism and the remodeling process. Markers of bone formation include procollagen type I N-terminal propeptide (P1NP), alkaline phosphatases (ALP), and osteocalcin (OCN), all of which reflect osteoblastic activity, while markers of bone resorption such as C-terminal telopeptide of type I collagen (CTX), N-terminal telopeptide (NTX), and tartrate-resistant acid phosphatase 5b (TRAP-5b) indicate osteoclastic bone degradation [29]. Among these, P1NP and CTX have been endorsed by international consensus as reference markers for evaluating bone turnover [30]. However, the clinical interpretation of BTMs in children is complicated by physiological variations associated with age, sex, and pubertal development, and reference intervals for pediatric populations remain limited and non-standardized across studies and geographic regions.

In the context of T1D, BTMs have emerged as useful tools for identifying early skeletal alterations that may not yet be apparent on imaging. Several studies have demonstrated that children and adolescents with T1D exhibit a pattern of low bone turnover, particularly characterized by reduced bone formation. A systematic review by Madsen et al. [31] encompassing 26 studies, found significantly lower OCN levels in youth with T1D compared to healthy controls, with a moderate inverse association with HbA1c, suggesting that poor glycemic control may suppress osteoblastic activity [31]. These findings were reinforced by a cohort study of 173 children with T1D by the same group, which reported decreased levels of OCN, P1NP, and CTX, independent of pubertal stage or disease duration [4]. Collectively, these findings suggest that chronic hyperglycemia and oxidative stress may disrupt normal bone remodeling in youth with T1D, contributing to an overall low bone turnover state. Given these observations, BTMs may serve as a valuable adjunct to traditional bone assessment methods in the early identification and monitoring of skeletal complications in this vulnerable population.

### 1.5. Fracture Risk

Impaired bone quality and quantity may translate to a higher fracture risk throughout the life span. A large population-based cohort study using data from The Health Improvement Network (THIN) in the United Kingdom evaluated 30,394 individuals aged 0–89 years with T1D and 303,872 individuals without T1D, matched for age, sex, and general practice [8]. The authors of the study reported elevated fracture risk across all age groups in individuals with T1D. The lowest hazard ratios (HR) were observed in males and females under the age of 20, with HRs of 1.14 (95% CI 1.01–1.29) and 1.35 (95% CI 1.12–1.63), respectively. The highest overall fracture risk was observed in men aged 60–69 years (HR 2.18, 95% CI 1.79–2.65) and women aged 40–49 years (HR 2.03, 95% CI 1.73–2.39). When examining hip fractures, the greatest risk was seen among men aged 60–69 years (HR 5.64, 95% CI 3.55–8.97) and women aged 30–39 years (HR 5.63, 95% CI 2.25–14.11) [8]. A meta-analysis of 39,925 adults with T1D aged 18–50 years further confirmed an increased fracture risk in young and middle-aged adults [32]. The authors of this meta-analysis reported a 1.9-fold increased risk of any fracture and a 4.4-fold increased risk of hip fracture compared to individuals without T1D. Several factors have been associated with increased fracture risk in this population, including poor glycemic control [8,33], the presence of microvascular complications [8], hypoglycemia events [34] and longer disease duration [35]. Bone fragility in individuals with T1D carries significant clinical implications, including increased morbidity, higher rates of hospitalization, reduced quality of life, and heightened healthcare costs [17]. The burden of fragility fractures in individuals with T1D is expected to rise, driven by both the increasing global incidence of the disease and improvements in life expectancy.

## 2. Prevention

**Figure 2:**
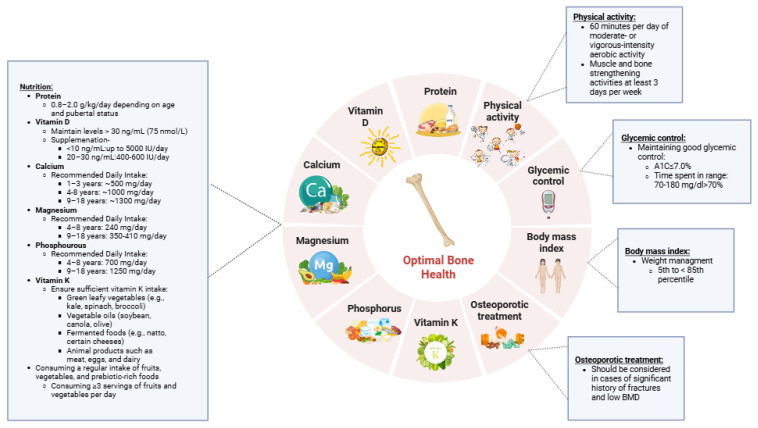
Recommendations for optimizing bone health in children and adolescents with type 1 diabetes.

Implementing targeted strategies to enhance skeletal health in children and adolescents with T1D is essential. Emphasizing optimal glycemic control, balanced nutrition, consistent physical activity, and maintaining a healthy weight can significantly reduce the lifelong risk of fractures (Figure 2).

## 3. Glycemic Control

Poor glycemic control, reflected by elevated HbA1c levels, has been linked to impaired bone accrual, reduced BMD, and increased fracture risk [36,37]. Weber et al. demonstrated that children with suboptimal glycemic control (HbA1c ≥ 7.5%) showed significantly reduced BMC accrual during the first year after diagnosis compared to those with better metabolic control [5]. In a cross-sectional study of 85 Danish children and adolescents with T1D, Fuusager et al. found that mean HbA1c levels were inversely associated with total body less head (TBLH) BMD Z-scores, even after adjusting for height and BMI. The negative association between glycemic control and BMD was statistically significant (β = −0.019, *p* = 0.012), emphasizing the adverse impact of chronic hyperglycemia on skeletal development [38]. Similarly, a large registry-based study by Eckert et al., involving 750 youth with T1D and fractures matched to 3750 T1D controls without fractures from the German/Austrian/Swiss DPV database, found that elevated HbA1c levels were significantly associated with an increased incidence of fractures [6]. Notably, this association was strongest in prepubertal males and postpubertal females, highlighting potential sex- and age-specific vulnerabilities to diabetes-related skeletal fragility [6].

Given the direct impact of hyperglycemia on bone health and the accumulating evidence linking poor glycemic control to an increased risk of fractures, improving glycemic control may reduce fracture risk in addition to mitigating other diabetes-related complications. The 2024 ISPAD guidelines recommend an HbA1c target of ≤6.5% (48 mmol/mol) for children and adolescents with access to advanced diabetes technologies, such as continuous glucose monitoring and automated insulin delivery [39]. In settings without these technologies, the target is ≤7.0% (53 mmol/mol). To achieve these HbA1c targets, it is recommended that individuals spend more than 70% of their time within the glucose range of 70–180 mg/dL [39].

## 4. Nutrition

Adequate nutrition during childhood and adolescence plays a crucial role in supporting optimal skeletal development [40]. A well-balanced diet that provides essential macronutrients—proteins, fats, and carbohydrates—and critical micronutrients such as calcium, phosphorus, and vitamin D is vital for bone formation and mineralization [41]. Although much of the current evidence arises from observational studies, the cumulative data underscore the significance of proper nutritional intake during skeletal growth to optimize bone strength and reduce future fracture risk [40].

Children and adolescents with T1D present unique nutritional challenges that must be addressed to support both metabolic regulation and bone health. As of today, dietary recommendations for youth with T1D are based on general healthy eating principles, with the ISPAD advising a macronutrient distribution of 45–50% carbohydrates, less than 35% fat (including <10% saturated fat), and 15–20% protein [42]. Available evidence suggests that dietary patterns among youth with T1D often deviate from recommended guidelines, characterized by insufficient consumption of fruits, vegetables, and whole grains, alongside excessive intake of processed foods and dietary fats compared to their healthy peers [43]. The SEARCH for Diabetes in Youth Study found that fewer than 40% of participants met targets for key nutrients, with food insecurity further impairing adherence to dietary guidelines [44]. These patterns may adversely affect both glycemic control and skeletal health, highlighting the need for structured nutrition education and targeted interventions to promote balanced eating habits and support optimal growth trajectories.

## 5. Protein

Protein intake plays a critical role in bone health by supporting bone matrix synthesis, enhancing muscle-mediated mechanical loading on bone, and stimulating anabolic pathways such as IGF-1 that promote bone formation and mineralization [20]. Inadequate protein intake has been shown to impair intestinal calcium absorption, potentially leading to compensatory elevations in parathyroid hormone (PTH) levels and increased bone resorption to maintain serum calcium homeostasis [41]. Data from the National Health and Nutrition Examination Survey (NHANES) revealed a positive association between higher protein intake and greater BMD at key skeletal sites, emphasizing the importance of adequate protein consumption for optimal skeletal accrual during adolescence [45]. This is further supported by findings from a 4-year prospective study in healthy children and adolescents aged 6–18 years, which demonstrated that long-term higher protein intake was significantly associated with greater periosteal circumference, cortical area, bone mineral content, and strength-strain index—markers indicative of improved bone size and mechanical strength [46]. In this cohort, protein intake averaged approximately 2 g/kg/day in prepubertal children and 1.5 g/kg/day during puberty [46].

Recent studies employing the indicator amino acid oxidation (IAAO) method—which is regarded as more accurate than traditional nitrogen balance techniques—suggest that current dietary protein recommendations may underestimate true physiological requirements during periods of growth. IAAO-based assessments indicate that the average protein requirement for school-aged children is approximately 1.3 g/kg/day, with a population-safe upper limit of around 1.55 g/kg/day—values that significantly exceed previous estimates [47,48].

In youth with T1D, reported protein intake generally aligns with or slightly exceeds international dietary recommendations, with mean contributions ranging from 16.8% to 21% of total energy intake [49,50]. However, despite appropriate protein consumption, children and adolescents with T1D exhibit decreased BMD and impaired bone health [50]. These findings suggest that while sufficient protein intake is important for general growth and bone matrix support, it may not be sufficient on its own to optimize bone mass accrual in youth with T1D, highlighting the multifactorial nature of bone health in this population. According to the ISPAD Clinical Practice Consensus Guidelines, children and adolescents with T1D should meet or modestly exceed standard protein intake recommendations (0.8–2.0 g/kg/day depending on age and pubertal status), ideally through nutrient-rich sources that support overall growth and metabolic health, while addressing additional risk factors that may compromise skeletal integrity [42].

## 6. Calcium

Calcium is the principal mineral component of bone, essential for skeletal rigidity and structural integrity. Inadequate calcium intake stimulates PTH secretion, promoting bone resorption and contributing to skeletal fragility [41]. Although maintaining sufficient calcium intake is critical during growth, particularly in vulnerable populations such as youth with T1D, achieving recommended levels remains a challenge. A cross-sectional study involving 122 adolescent girls aged 10–16 years, including 62 with T1D and 60 healthy controls, assessed dietary intake, bone structure, and biochemical markers of bone health. The mean daily calcium intake was 950 ± 410 mg in the T1D group and 862 ± 357 mg in the control group, with both values falling below the Recommended Dietary Allowance (RDA) of 1300 mg/day for this age group [51]. Similarly, a large cross-sectional analysis of 238 youth with T1D found that approximately one-third failed to meet calcium intake recommendations, with female sex and obesity emerging as significant risk factors for insufficient intake [52]. Beyond dietary intake, disturbances in calcium metabolism have also been implicated in impaired bone health in T1D. In a longitudinal study, greater urinary calcium excretion was independently associated with diminished bone accrual over a 12-month period in youth with T1D, independent of calcium intake, body size, or vitamin D status, suggesting that hypercalciuria—potentially related to altered bone turnover or dysregulated PTH response—may contribute to suboptimal skeletal development [53]. This study demonstrated a positive correlation between urinary calcium excretion and hyperglycemia [53]. Together, these findings underscore the importance of both optimizing calcium intake and addressing calcium losses to support bone health and reduce fracture risk in youth with T1D.

Dietary intake can be increased by prioritizing calcium-rich foods such as dairy products (milk, yogurt, cheese), fortified plant-based alternatives, leafy green vegetables, and fortified cereals [41]. Additionally, calcium-rich mineral waters, particularly those high in bicarbonates, offer excellent calcium bioavailability comparable to dairy products [54]. When dietary intake is insufficient or adherence is challenging, calcium supplementation should be considered. Calcium carbonate is the preferred supplement due to its high elemental calcium content (40%), cost-effectiveness, and good absorption when taken with food. The recommended dose of calcium supplementation for children is 25–75 mg/kg/day of elemental calcium, given in 2–3 divided doses [55]. Evidence from a randomized controlled trial by Khadilkar et al. supports the efficacy of both dairy-based and pharmacological calcium supplementation in improving bone health, with calcium carbonate offering a practical option, particularly in resource-limited settings [56]. Given the increased risk of impaired calcium metabolism and urinary calcium losses in youth with T1D, early nutritional intervention is essential to support bone development and reduce fracture risk.

## 7. Vitamin D

Vitamin D plays a central role in skeletal health by enhancing intestinal calcium and phosphorus absorption and ensuring the proper mineralization of the bone matrix [57]. Vitamin D deficiency is highly prevalent among children and adolescents with T1D, potentially contributing to impaired bone development. A recent systematic review and meta-analysis of 45 studies involving 6995 youth with T1D reported a global vitamin D deficiency prevalence of 45%, with an additional 33% classified as insufficient and only 27% achieving sufficient levels [58]. Similarly, a cross-sectional study involving 453 Indian youth with T1D demonstrated a mean serum 25-hydroxyvitamin D level of 20.4 ± 11.3 ng/mL, with 24.5% deficient, 31.1% insufficient, and 44.4% sufficient [59], based on Endocrine Society criteria [60]. In the same cohort, vitamin D concentrations showed a significant positive correlation with trabecular bone density measured by pQCT. Additionally, poorer glycemic control, higher diastolic blood pressure, and female sex were associated with increased risk of deficiency [59].

Animal studies provide additional insights, suggesting that active vitamin D (1α,25-dihydroxyvitamin D_3_) may protect against diabetes-induced bone loss by enhancing osteoblast function, reducing oxidative stress, and suppressing FoxO1-mediated autophagy. These preclinical findings offer potential mechanisms by which vitamin D could support bone health in human youth with T1D [61].

Treatment strategies aimed at correcting calcium and vitamin D deficiencies have shown promising effects on bone health in youth with T1D. A randomized controlled trial of 203 underprivileged Indian youth with T1D compared daily supplementation with either 200 mL of cow’s milk plus 1000 IU of vitamin D_3_ or 500 mg of calcium carbonate plus 1000 IU of vitamin D_3_ to standard care. Both supplementation groups experienced significant improvements in lumbar spine BMD Z-scores and cortical thickness, particularly in girls, whereas the control group showed no improvement and experienced a decline in cortical thickness [56].

Strategies to elevate vitamin D levels through dietary sources include regular consumption of fatty fish such as salmon, mackerel, and sardines, which are naturally high in vitamin D_3_. Fortified foods, including milk, orange juice, cereals, dairy products, and egg yolks, provide additional dietary vitamin D. Mushrooms exposed to UV light and cod liver oil supplements are also beneficial [62]. All children should meet their recommended daily intake of vitamin D—at least 600 IU (15 micrograms)—through diet and/or supplementation, as advised by the Institute of Medicine (IOM) [63]. This is especially important for children with limited sun exposure. Routine screening of 25-hydroxyvitamin D levels is recommended for children with type 1 diabetes.

## 8. Magnesium

Magnesium is an essential mineral for maintaining bone health, regulating bone mineralization, influencing osteoblast and osteoclast function, and stabilizing the bone matrix [64]. Recent studies highlight mineral metabolism disturbances in children and adolescents with T1D. Serum magnesium levels were found to be significantly lower in youth with T1D (0.86 ± 0.07 mmol/L) compared to healthy peers (0.91 ± 0.06 mmol/L; *p* = 0.005), and this was associated with poor glycemic control [65]. Similarly, an observational cohort of 71 children with T1D (mean age 9.68 ± 3.99 years) reported hypomagnesemia in 28.2% of participants, with mean serum magnesium levels of 1.83 ± 0.27 mg/dL [66]. Hypomagnesemia was more evident in patients with poor diabetic control and those with higher atherogenic lipid parameters [66].

## 9. Phosphorus

Phosphorus plays a vital role in skeletal development as a structural component of hydroxyapatite and is essential for bone mineralization [64]. Dietary phosphorus deficiency is rare in healthy individuals, as phosphorus is widely available in protein-rich foods such as dairy products, meat, grains, legumes, nuts, and is also commonly present in processed foods and food additives [67]. However, phosphorus metabolism may be altered under certain physiological conditions. For example, adolescents undergoing rapid growth have increased requirements, with a recommended daily allowance (RDA) of 1250 mg to support optimal bone development [68]. A cross-sectional study comparing 34 children with T1D to 17 healthy controls found no significant difference in dietary phosphorus intake between groups (1382.7 mg/day vs. 1248.7 mg/day, *p* = 0.68), yet serum phosphorus levels were significantly lower in the T1D group (4.4 ± 0.8 mg/dL vs. 4.9 ± 0.7 mg/dL, *p* = 0.03) [50].

## 10. Vitamin K

Vitamin K is essential for bone health, primarily through its role in activating vitamin K-dependent proteins such as osteocalcin and matrix Gla-protein [69]. Osteocalcin promotes bone mineralization by binding calcium, enhancing BMD and reducing fracture risk, while matrix Gla-protein inhibits vascular calcification, indirectly supporting bone quality [70]. Clinical and epidemiological evidence links adequate vitamin K status with increased BMC and a lower risk of low-energy fracture [71].

In pediatric populations, vitamin K deficiency, often subclinical, can adversely affect bone health, potentially increasing susceptibility to fractures [72]. Although general evidence supports the importance of adequate vitamin K for bone health in children, specific data regarding children and adolescents with T1D remain sparse. Given that children with T1D inherently possess increased risk factors for reduced BMD and skeletal fragility, more targeted research is necessary to determine the efficacy and safety of vitamin K supplementation in this subgroup.

To ensure sufficient vitamin K intake in children with T1D, dietary strategies should emphasize green leafy vegetables (e.g., kale, spinach, broccoli), vegetable oils (soybean, canola, olive), fermented foods (e.g., natto, certain cheeses), and animal products (meat, eggs, and dairy). Regular consumption of these vitamin K-rich foods may help optimize bone mineralization and overall skeletal health in children, particularly those with type 1 diabetes, although precise dietary guidelines remain to be established pending further research.

## 11. Fruits, Vegetables, and Prebiotics

A growing body of evidence highlights the importance of fruits and vegetables in supporting bone health due to their high content of essential micronutrients, including vitamins, antioxidant-rich phytochemicals, and dietary fiber [73,74]. These components have been shown to support bone health by modulating oxidative stress. Prebiotic fibers such as fructo-oligosaccharides (FOS), inulin, and galacto-oligosaccharides (GOS) undergo fermentation in the colon, resulting in the production of short-chain fatty acids like acetate, propionate, and butyrate, which enhance calcium absorption by lowering intestinal pH and promoting beneficial gut microbiota diversity [75,76]. Clinical studies in adolescents have demonstrated that supplementation with 8 g/day of FOS and inulin over one year improved whole-body BMC, while short-term intake of 2.5–5 g of GOS twice daily increased fractional calcium absorption and fecal bifidobacteria levels [77,78]. In adolescents, supplementation with 8 g/day of FOS and inulin for one year has been shown to improve whole-body BMC. Similarly, 15 g/day of oligofructose increased fractional calcium absorption. In a study involving healthy girls aged 10–13 years, short-term supplementation with 2.5–5 g of GOS twice daily significantly increased calcium absorption, especially in the distal intestine, and was accompanied by an increase in fecal bifidobacteria—supporting the hypothesis that changes in the microbiota mediate this effect [77].

Potassium, abundant in fruits and vegetables in the form of organic salts such as citrate and malate, plays a supportive role in skeletal health by helping to neutralize dietary acid load. This buffering capacity reduces the reliance on bone-derived alkaline reserves, thereby decreasing calcium mobilization from bone and urinary calcium excretion [79,80,81]. Chronic low-grade metabolic acidosis—commonly associated with Western-style diets low in potassium—has been shown to stimulate osteoclast activity and inhibit osteoblast function, leading to increased bone resorption [79,82,83]. In vitro studies further suggest that potassium citrate may directly inhibit osteoclastogenesis while promoting osteoblast proliferation and alkaline phosphatase activity, highlighting its potential in supporting bone integrity [83].

Observational data further support the association between fruit and vegetable intake and skeletal outcomes. Consuming ≥3 servings of fruits and vegetables per day during adolescence has been associated with larger bone size, while intake of ≥5 servings per day in adults correlates with higher BMD and reduced hip fracture risk [84,85].

In children and adolescents with T1D, higher intake of fruits and vegetables was positively associated with BMD and BMC [50]. Similarly, Thongpaeng et al. emphasized the need for tailored nutritional counseling to promote increased fruit and vegetable consumption in this population [49]. Given the documented suboptimal dietary habits in youth with T1D, strategies that emphasize the regular intake of fruits, vegetables, and prebiotic-rich foods may offer a feasible and effective approach to support bone development and reduce long-term fracture risk. Healthcare professionals should proactively address these concerns by providing personalized dietary guidance that balances glycemic control with nutritional adequacy. Structured education on portion sizes, meal timing, and carbohydrate pairing can support patients in safely incorporating a wider variety of nutrient-rich foods—including fruits and vegetables—into their diets, which are essential for bone health.

## 12. Physical Activity

Levels of physical activity in children and adolescents with T1D are consistently lower than in their healthy peers. According to a meta-analysis, youth with T1D perform approximately 13 min less moderate-to-vigorous activity per day and spend over an hour more in sedentary behaviors compared to non-diabetic controls [86]. Additionally, data from a cross-sectional study show that only about one in four children with T1D meet the daily recommendation of 60 min of physical activity [87]. This reduced engagement is largely due to barriers such as concerns about hypoglycemia, challenges in managing glucose levels around exercise, low physical fitness, and lack of adequate support from caregivers and peers [86,87,88].

Physical activity—particularly weight-bearing exercises, such as jumping, running, or ball games—has been shown to improve BMD in this population. A randomized controlled trial demonstrated that nine months of regular weight-bearing exercise led to significant increases in BMD among children with T1D, achieving improvements comparable to those seen in healthy peers [89]. A systematic review further confirmed the benefits of structured exercise on bone accrual in this group [90]. Moreover, recent data suggest that physical activity may attenuate differences in bone microarchitecture between children with and without T1D [91], and is associated with improvements in bone microarchitecture among young adults [92].

These findings highlight the need to promote safe, consistent, and supervised physical activity to support optimal skeletal development in children and adolescents living with T1D. The American Diabetes Association recommends that children and adolescents with T1D follow the general physical activity guidelines, which include 60 min per day of moderate- or vigorous-intensity aerobic activity, along with muscle-strengthening and bone-strengthening activities at least three days per week [93]. This approach is essential not only for overall metabolic and psychological health but also for optimizing bone health.

## 13. Body Mass Index (BMI)

The American Diabetes Association (ADA) recommends that osteoporosis and fracture prevention in individuals with T1D align with general population guidelines for maintaining a healthy weight [94]. Accordingly, a BMI between the 5th and 85th percentiles for age and sex is considered optimal for bone health in the pediatric population.

## 14. Monitoring BMD and Antiresorptive Medications

The ISPAD does not recommend routine DXA screening in youth with T1D, except in the presence of comorbid conditions such as celiac disease [1]. The ADA advises that the use of antiresorptive therapy should follow the same guidelines applied to the general population [94]. However, such treatment is rarely indicated in children and adolescents, and is typically reserved for those with confirmed osteoporosis, as defined by the International Society for Clinical Densitometry (ISCD)—specifically, individuals with a history of low-trauma vertebral fractures or multiple long bone fractures accompanied by a BMD Z-score below −2 [95].

## 15. Conclusions

Children and adolescents with T1D frequently exhibit alterations in bone health, characterized by reduced BMD, impaired bone microarchitecture, and decreased bone turnover compared to their healthy counterparts. These skeletal abnormalities contribute to an elevated lifetime risk of fractures in this population. Optimizing skeletal health in the pediatric population with T1D requires a multifaceted approach encompassing stringent glycemic control, adequate nutritional intake, and the promotion of regular physical activity. Emerging evidence highlights the synergistic effects of these proposed strategies in mitigating the heightened lifetime risk of fractures observed in individuals with T1D. Healthcare providers, including physicians and dietitians, must be well-versed in these strategies, actively counsel patients and their families, and diligently monitor adherence to ensure effective implementation.

## Abbreviations

T1D-type 1 diabetes, BMD-bone mineral density, BMC-bone mineral content, AGEs-advanced glycation end products, GH-growth hormone, IGF-1-insulin-like growth factor-1, DXA-dual-energy X-ray absorptiometry, pQCT-peripheral quantitative computed tomography, QUS-quantitative ultrasound, BTMs-bone turnover markers, ALP-alkaline phosphatases, OCN-osteocalcin, CTX-C-terminal telopeptide of type I collagen, NTX-N-terminal telopeptide, TRAP-5b-tartrate-resistant acid phosphatase 5b, RDA-recommended dietary allowance, GOS-galacto-oligosaccharides, FOS-fructo-oligosaccharides, P1NP-procollagen type I N-terminal propeptide, TBLH-total body less head.
